# Are poor set-shifting and central coherence associated with everyday function in anorexia nervosa? A systematic review

**DOI:** 10.1186/s40337-021-00392-x

**Published:** 2021-03-29

**Authors:** Kelly M. Dann, Phillipa Hay, Stephen Touyz

**Affiliations:** 1 School of Psychology, University of Sydney, Sydney, Australia; 2 School of Medicine, Western Sydney University, Sydney, Australia; 3 InsideOut Institute and School of Psychology, University of Sydney, Sydney, Australia

**Keywords:** Anorexia Nervosa, cognition, set-shifting, cognitive flexibility, central coherence, executive function, functional outcome, neuropsychology, quality of life, eating disorders

## Abstract

**Background:**

There is increasing interest in associations between cognitive impairments and clinical symptoms in Anorexia Nervosa (AN), however, the relationship with everyday function is unclear. The current review synthesizes existing data regarding associations between scores on tests of set-shifting and central coherence and functional outcome measures for individuals with AN.

**Method:**

A systematic electronic database search yielded 13 studies which included participants with current or lifetime AN where scores on a neuropsychological test of set-shifting or central coherence were directly or indirectly compared to a functional outcome measure.

**Results:**

Associations between set-shifting and central coherence performance measures and functional outcomes were limited in number and noted only in adult or mixed-age cohorts. Associations were noted at subscale level, suggesting they are specific in nature. In younger cohorts, assessments of executive functioning in everyday life appear sensitive to cognitive-behavioral flexibility issues.

**Conclusions:**

Associations between cognitive performance and functional outcome have not been as systematically assessed in AN as in other psychiatric disorders. Key factors to address in future research include: (a) the use of function measures which are sensitive to both the level of impairment, and specific rather than general impairments (b) the ecological validity of measures, (c) the task impurity problem, especially in regard to cognitive flexibility assessment, and (d) the need to measure both cognitive deficits and strengths*,* because tests of specific cognitive processes may underestimate the ability to function in daily life due to compensatory strategies.

## Plain English summary

The current review gathers existing evidence regarding whether poor performance on tests of cognitive flexibility and detail-oriented processing are associated with difficulties in everyday function for people with Anorexia Nervosa. A literature search identified 13 articles which included data which address this question. The associations between poor performance on cognitive tests and day-to-day function were not strong, and were noted only in adult and mixed-aged groups. In younger samples, self-report measures of everyday cognitive skills appear to be more sensitive to function issues. Overall this review suggests that more research is needed to better understand how group differences on cognitive flexibility and detail-oriented processing tests relate to daily functioning for individuals with Anorexia Nervosa. It is recommended that future research should also measure cognitive strengths which may be used to compensate for cognitive weaknesses in everyday life.

## Background

Anorexia Nervosa (AN) is an eating disorder (ED) characterized by extreme dietary restriction and other weight-loss behaviors, and a persistent fear of weight-gain despite significantly low weight [1]. The complex and serious physical and psychological health issues associated with AN result in significant health-related disability. Individuals with AN experience severe impairment in their social and occupational function [83], and longitudinal research highlights poor psychosocial functioning as a significant predictor of mortality [30].

In the past 10 years there has been increasing interest in neurocognitive functioning in ED research, with a recent ‘review of reviews’ finding 28 systematic and meta-analytic publications since 2010 [73]. A consistent finding in the literature is that adults with AN perform more poorly on neuropsychological tests of set-shifting and central coherence than healthy control groups [45, 100]. Set-shifting is the ability to shift focus between mental sets in response to changing demands, and is a measure of cognitive flexibility [21]. Central coherence refers to the normal tendency to process information in a global or holistic manner, rather than with excessive attention to detail. In contrast, a focus on detail at the expense of overall conceptual and contextual understanding, is an attentional bias known as weak central coherence [37]. Poor performance in these two areas forms a cognitive profile of inflexible, detail-focused processing which is consistent with clinical observations of cognitive-behavioral rigidity in individuals with AN. The Cognitive-Interpersonal Maintenance Model for AN [72] proposes poor set-shifting and weak central coherence underlie obsessive-compulsive traits, and together with socio-emotional avoidance, are key risk factors in both the aetiology and maintenance of the disorder. This inflexible cognitive style is further suggested to contribute to poor treatment engagement and response [84].

Taking a broader perspective, deficits in set-shifting and/or central coherence are present in other psychiatric disorders, prompting suggestions that the impairments may be a trans-diagnostic marker, or general risk-factor for a range of disorders. Within this wider psychiatric literature, cognitive deficits across the domains of attention, memory and executive function have been shown to predict both clinical and functional outcomes. Meta-analytic results suggest a substantial effect of general cognitive deficits on overall functional outcome in Schizophrenia [34], Bipolar Disorder [20], and a more limited general effect in Major Depressive Disorder [26]. In AN there has been no synthesis of the existing evidence of the effect of cognitive impairments on functional outcomes. However, a substantial body of research has focused on the measurement of cognitive impairments and possible associations with clinical characteristics of the disorder.

### Set-shifting and central coherence impairments in AN

#### Set-shifting

A comprehensive meta-analysis which included 1394 participants with AN [100], found set-shifting deficits across a range of tasks of small to medium effect (Hedges' *g* = − 0.44). The effect was not consistent across included studies; 29 of 49 studies did not find significant differences between AN participants and healthy control cohorts. Effect sizes also varied greatly by measure; performance was most impaired in the Haptic Illusion task [87] (*g* = − 1.02), but not significantly different to controls in the Intra-Extra Dimensional Set Shift test [71] (*g* = − 0.17). There are suggestions that shifting is not impaired in all domains; participants with AN have demonstrated superior category switching compared to controls on tests of verbal fluency [77, 78].

Set-shifting is most commonly assessed using the Wisconsin Card Sort Task (WCST; [32]) on which individuals with AN often perform poorly. A meta-analysis of 22 studies which assessed set-shifting using the WCST found significant effect for adults (Cohen's *d* = .48) but not children (*d* = .25) [95]. This is consistent with meta-analysis across a range of executive function tasks which found effects were not significant for children with AN [40], however other meta-analytic results have not yielded significant differences between adolescent and adult participants [100] suggesting further clarification is needed.

Differences in shifting ability between AN Restricting subtype (AN-R) and Binge-Purge subtype (AN-BP) are inconsistent [73], however studies are often limited in their power to detect effects by sub-type. Splitting by sub-type, meta-analytic results of 11 studies including participants with AN-R suggest set-shifting deficits of medium effect (*g* = − 0.51) but across 6 studies the effect size for AN-BP was not significant (*g* = − 0.18) [100]. Overall, the literature suggests the presence of set-shifting impairments, but effects are not consistent, and vary greatly by measure.

#### Weak central coherence

Meta-analytic results of 7 studies using the Rey Osterreith Complex Figure Test (ROCFT; [61]) in AN indicate difficulties with global processing of medium to large effect (*d* = 0.63) [45, 47]. A synthesis of 5 studies using the Group Embedded Figures Test (GEFT; [98, 99]) shows a bias towards local processing, also of medium to large effect (*d* = 0.63) [45, 47]. Taken together, these results indicate weak central coherence in participants with AN. Significant differences in central coherence by AN subtype have been suggested, with AN-R participants noted to perform more poorly than AN-BP on the Wechsler Block Design and Object Assembly tasks [88], however the validity of the Block Design task as a central coherence measure has been questioned [45, 47, 50]. A meta-synthesis of studies using the ROCFT found participants with AN, and a group of unaffected relatives of AN patients both scored significantly lower on the central coherence index than healthy controls, but participants recovered from AN performed similarly to controls [46]. These findings are difficult to interpret, since poor performance by unaffected relatives suggest deficits may be trait-based, while similar performance to controls by the recovered group suggests issues may be state-dependent.

### Associations between cognitive impairments and clinical characteristics of AN

Associations between cognitive impairments and clinical characteristics of the disorder provide support for the Cognitive-Interpersonal Maintenance Model for AN [72]. Poor set-shifting has been associated with a longer duration of illness, more severe ED rituals [69] and illness severity [84]. Weak central coherence has been associated with severity of illness, BMI and ED-related compulsions [70]. However, evidence regarding the relationship between cognitive impairments and clinical characteristics is quite inconsistent, which has prompted suggestions that deficits may be “clinically silent” [67]. The direction of causality is also difficult to interpret since cognitive flexibility is impacted by even short-term starvation in healthy participants [8, 63].

Cognitive Remediation Therapy (CRT) for AN is a treatment augmentation which aims to improve set-shifting and central coherence [81]. Since difficulties in these areas are thought to be both a risk factor in the maintenance of the disorder and also a barrier to engagement with treatment, remediation may improve clinical outcomes. Early reviews showed encouraging results and good patient acceptance in both adult and adolescent cohorts [82, 85]. However, a recent meta-analysis of randomized controlled trials of CRT versus control treatments showed no improvement in central coherence, ED symptoms or BMI, and mixed results for set-shifting which were unable to be synthesized due to the wide variety of measures used [35]. These results require cautious interpretation; since CRT is a relatively new intervention they include participants across a wide age group and both AN subtypes. Part of the CRT program involves applying new thinking styles to everyday life, and two studies which have included quality of life (QoL) outcomes [22, 90] have shown CRT is associated with improvement in ED-related QoL [35].

### Functional outcome in AN

Functional impairment, or limitation in ability in areas of everyday living such as social, academic and occupational function, does not form part of the diagnostic criteria of AN. However, improvement in function is widely acknowledged as a key marker of recovery, and a self-report measure of function or QoL is often included as a secondary outcome in empirical research.

The Clinical Impairment Assessment (CIA; [10]), is a widely-used ED-specific self-report measure designed to assess functional impairments secondary to eating disorder symptoms. Weight and shape concerns and binge eating frequency have been shown to significantly predict CIA scores [66], and impairment on the CIA has also been demonstrated to increase as a function of illness severity according to BMI [18]. A recent network analysis of data from participants with AN found moderate correlations between ED symptoms at baseline and post-treatment CIA scores [24].

Results from assessments of QoL in AN are somewhat mixed, and may be complicated by the ego-syntonic nature of the disorder, wherein aspects of the disorder are perceived as congruent with the ideal self. Participants with AN-R have been noted to score similarly to healthy controls on a subjective QoL scale, possibly because items may be interpreted differently by AN patients whose concerns are not associated with low weight, and where weight increases towards a healthy range may be associated with a reduction in QoL ratings [58]. Mixed results appear to be more associated with shorter term follow-up measures, and shorter duration of illness [55], but in participants with severe and enduring AN, a close association between QoL, BMI and ED symptom severity has been demonstrated [5].

Functional impairment in AN is most commonly assessed as a secondary outcome, however longitudinal research has demonstrated that health-related QoL has a bi-directional relationship with ED symptoms - lower QoL scores predicted increases in ED symptoms over periods up to 4 years - suggesting QoL is a valid treatment target [55]. QoL, mood and social adjustment were the primary outcome measures in a randomized controlled trial of two alternative treatments for severe and enduring AN which demonstrated an unusually high retention rate, suggesting the approach may also increase patient engagement [86]. As mentioned earlier, CRT has been associated with preliminary, yet encouraging improvements in self-reported QoL in two studies [35]. These results suggest QoL could be more broadly explored as a treatment target in future CRT interventions.

### The current review

Understanding the possible impact of cognitive factors on everyday function is important because functional improvements are milestones of recovery. Cognitive Remediation Therapy (CRT) is being implemented in AN, and it has been suggested that further evaluation of CRT should include whether it supports improvement in everyday functioning [11, 49]. To support the inclusion of broader functional outcomes in the evaluation of current therapies, a clearer understanding of the impact of cognitive inefficiencies on day-to-day function in AN is needed. To address this need, we conducted a systematic review of the literature to answer the question: Are poor set-shifting and central coherence associated with everyday function in Anorexia Nervosa?

## Method

### Inclusion and exclusion criteria

Inclusion criteria for studies were:

- Participants met the criteria for current or lifetime Anorexia Nervosa.
- Comparison between scores on a neuropsychological test of set-shifting or central coherence and scores on a measure of everyday function, or concurrent measurement of these variables compared to a healthy control group was reported.

A broad definition of everyday function which included social function, quality of life scales and measures of cognitive flexibility or detail-oriented processing in everyday life was used to maximize the breadth of studies included. We did not apply any age limit on participants because studies in AN often include a wide age range to maximize power.

### Literature search

A search was conducted in November 2019 and updated in February 2021. Embase, Medline, PsychInfo and Scopus databases were searched using the following general and Medical Subject Headings (MeSH) search terms:

“anorexia nervosa” AND.

“daily function” OR “quality of life” OR “activities of daily living” OR “functional outcome” OR “employment” OR “independent living” OR “work and social adjustment scale” OR “Clinical impairment assessment” OR “eating disorders quality of life” OR “inventory of interpersonal problems” OR “Health related quality of life in eating disorders” OR “SF-36” OR “social function” OR “psychosocial function” OR “socio-emotional function” OR “social cognition” OR “ecological validity” OR “ecologically valid” OR “Behavioural assessment of dysexecutive syndrome” OR “Behaviour rating inventory of executive function” OR “detail and flexibility questionnaire” OR “D-Flex” AND.

Neurocogniti* OR neuropsych* OR “cognitive assessment” OR “neuropsychological assessment” OR “cognitive style” OR “cognitive deficit” OR “cognitive flexibility” OR “executive function” OR “ set shifting” OR “task switching” OR “central coherence” OR “Rey-Osterreith” OR “Wisconsin card sort” OR “Group embedded figures” OR “trail making” OR “Fragmented pictures” OR “Hayling” OR “Brixton” OR “Ravello Profile” OR “Delis Kaplan” OR “NEPSY*”.

Initial results were limited to English language, human subjects and peer-reviewed articles where possible. The database search was supplemented by a ProQuest Dissertations and Theses search using the above terms to search the full bibliographic record excluding the full text (NOFT), and a manual reference list search to identify any further articles relevant to the review. The review was performed according to the Preferred Reporting Items for Systematic reviews and Meta-Analyses (PRISMA) standards. Two reviewers (K.D. & S.T.) independently reviewed a subset of the articles at both abstract and full-text stage to determine eligibility.

## Results

**Fig. 1:**
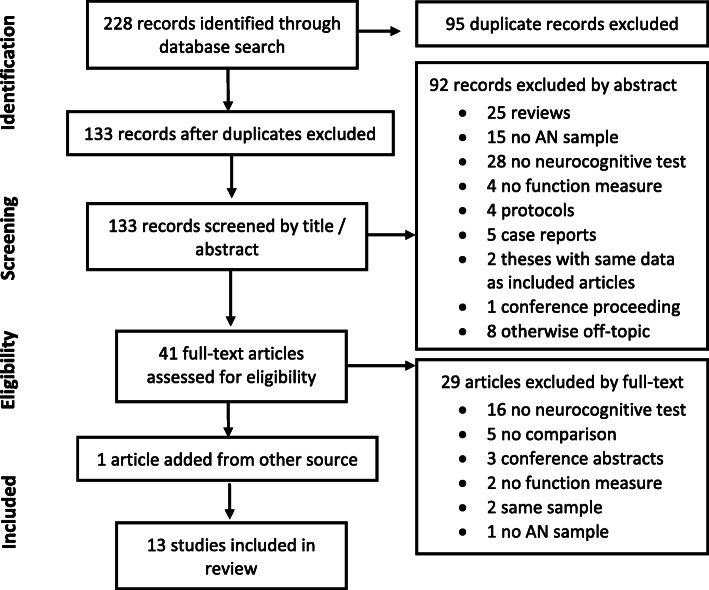
PRISMA flow diagram for study selection

Thirteen articles met the inclusion criteria. The systematic search process is illustrated in Fig. 1. Included studies used a variety of measures of set-shifting, central coherence and functional outcome, and different methods of analysis, therefore a descriptive review was conducted. The included studies are summarized in Table 1. The table is organized according to the type of functional outcome measure used. The first section includes studies which used general function, social function and quality of life measures. The second section includes studies which employed measures of executive functioning in daily life.

**Table 1:** Summary of studies included in the review

*Author (year)*	*Sample*	*Cognitive measures*	*Function measures*	*Results*
Oldershaw, Lavender & Schmidt [60]	AN (*n* = 71) Outpatients. Age over 18 years, *M* age = 26	WCST, Brixton, TMT, GEFT	CIA	Cognitive tests did not predict CIA scores.
Bentz et al. [9]	AN (*n* = 71) 43 first episode *M *age = 16, 28 recovered *M *age = 18. HC (*n* = 41) Age 14–22 years.	D-KEFS: Composite set-shifting measure (shift conditions of Verbal Fluency, Design Fluency and Trail Making), GEFT	ADOS	Composite SS and GEFT did not predict ADOS social function.
Hamatani et al. [36]	AN (*n* = 21: AN-BP = 13, AN-R = 8). 14 outpatients, 7 inpatients. Adults, *M* age = 32 years. HC (*n* = 25).	KWCST, ROCFT	SF-36	Greater KWCST DMS associated with lower scores on SF-36 PCS (*r* = − 0.658, *p* = < .05). ROCFT CCI 30-min delay recall score associated with SF-36 MCS (*r* = 0.610, *p* = < .05).
Westwood, Mandy & Tchanturia [94]	AN (*n* = 99) Inpatients and outpatients. Age 12–47 years.	WCST, ROCFT, D-Flex	ADOS	Greater WCST %PE associated with increased ASD symptoms, Welch’s *F* (2,59.77) = 3.77, *p* = .029. D-flex increase in cognitive rigidity scores between no vs. high ASD symptoms, *p* = .018 ROCFT not associated with increased ASD symptoms.
Renwick et al. [67]	AN (*n* = 100; AN-R = 44, AN-BP = 33, EDNOS-AN = 23) Outpatients. Adults, *M* age = 24.7 years.	WCST, Brixton, ROCFT	CIA Reading the Mind in Films Task	Cluster (17% of participants) show poor performance on WCST, ROCFT CCI and RMIF emotional ToM. No effect of cluster for ROCFT, but overall poor performance. No effect of cluster for CIA.
Talbot, Hay & Touyz [79]	AN (*n* = 49; 24 acute, 10 weight-recovered, 15 fully recovered) HC (*n* = 43) Age 19–27 years.	WCST, ROCFT, MFFT	EDQOL, IIP-32	WCST PE higher scaled scores (better performance) associated with higher IIP-32 ‘Too dependent’ (*r* = .305, *p* = .033), ‘Too aggressive’ (*r* = .401, *p* = .004), ‘Hard to be involved’ (*r* = .313, *p* = .029) and ‘Hard to be supportive’ (*r* = .294, *p* = .040). WCST CC associated with higher IIP-32 ‘Too dependent’ (*r* = .354, *p* = .013), ‘Too aggressive’ (*r* = .334, *p* = .019) and better financial QOL (*r* = −.393, *p* = .007). ROCFT lower CCI and OCI associated with higher IIP-32 ‘Hard to be supportive’ (CI: *r* = −.336, *p* = .020; OCI: (*r* = −.413, *p* = .004) and lower OCI associated with higher IIP-32 ‘Hard to be involved’ (*r* = −.343, *p* = .017).
Calderoni et al. [14]	AN-R (*n* = 23) HC (*n* = 46) Age 9–16 years.	NEPSY-II Attention and EF: Auditory response set, Inhibition shifting	NEPSY-II Social Perception: ToM verbal, ToM contextual and Affect recognition	NEPSY-II Attention and EF no group differences on overall domain but response set shifting test worse in AN vs. HC (*t*_67_ = − 2.17, *p* = .033). No group differences on NEPSY-II inhibition shifting or NEPSY-II social perception domain.
Harrison, Tchanturia, Naumann & Treasure [38]	AN (*n* = 85; 35 AN-R, 15 AN-BP, 35 recovered) HC (*n* = 90) Age 18–55 years.	WCST, Brixton, ROCFT, GEFT, FPT	Reading the Mind in the Eyes test	Higher WCST PE associated with lower Reading the Mind in the Eyes emotion recognition (*r*_s_ = −.281, *p* = .05), but not significant after Bonferroni correction.
**Executive function in daily life**				
Spitoni, Aragonaa, Bevacqua, Cotugno & Antonucci [75]	AN-R (*n* = 62) HC (*n* = 70) Age 16–42 years.	WCST, TMT, ROCFT	BADS	AN WCST PE higher (partial *ƞ*^2^ = .03) and TMT-B slower (partial *ƞ*^2^ = .10) than HC. AN lower ROCFT CCI than HC at 30s (partial *ƞ*^2^ = .13) and 20 min recall (partial *ƞ*^2^ = .11). AN BADS accuracy similar, but AN slower than HC across tasks (Rule shift cards time: partial *ƞ*^2^ = .14; Key search time: partial *ƞ*^2^ = .83, Zoo map planning time: partial *ƞ*^2^ = .18; Zoo Map execution time partial *ƞ*^2^ = .12)
Herbrich, Kappel, van Noort & Winter [39]	AN (*n* = 111; AN-R = 90, AN-BP = 21) HC (*n* = 63) Age 9–17 years.	WCST, TMT, ROCFT, GEFT	BRIEF-SR	AN-R TMT-4 faster than HC (*d* = .39) No group differences for WCST, ROCFT or GEFT. BRIEF-SR: ‘Shift’ subscale both subtypes higher than HC (AN-R; *d* = −.89) and (AN-BP; *d* = − 1.41) and AN-BP higher than AN-R (*d* = .57).
van Noort, Kraus, Pfeiffer, Lehmkuhl & Kappel [89]	AN (*n* = 20) 15 inpatients, 5 outpatients. HC (*n* = 20) Age 12–18 years.	TMT, ROCFT	BRIEF-SR	No group differences for TMT or ROCFT at baseline. Irrespective of time point, AN less flexible than HC on BRIEF-SR subscales ‘Cognitive shift’ (partial *ƞ*^2^ = −.207), ‘Behavioral shift’ (partial *ƞ*^2^ = −.230).
Stedal & Dahlgren [76]	AN (*n* = 20, AN-R = 18, AN-BP = 2). 10 in-, 10 outpatients Age 13–18 years.	D-KEFS: TMT-4, Color-word interference test-4 and verbal fluency-3, Brixton, ROCFT	BRIEF-SR BRIEF-PF	All neurocognitive test scores, BRIEF-SR and BRIEF-PF were within normal range.
McAnarney et al. [54]	AN-R (*n* = 24) HC (*n* = 37) Age 14–20 years.	WCST, CANTAB ID/ED	BRIEF-SR BRIEF-PF	AN-R no significant differences in WCST PE or CANTAB ID/ED. AN-R greater set-shifting difficulties than HC on BRIEF-PF (*M* = 54.5 vs. 47, *p* = <.008) and BRIEF-SR (*p* = .057). BRIEF-SR ‘Behavioral Shift’ sub-scale scores show larger difference (*M* = 64.1 vs. 48.5, *p* = <.008) than ‘Cognitive Shift’ (*M* = 54.9 vs. 48.7, *p* = <.008).

*Abbreviations*: *ADOS* Autism Diagnostic Observation Schedule, *BADS* Behavioral Assessment of Dysexecutive Syndrome, *BRIEF-PF* Behavior Rating Inventory of Executive Function Parent Form, *BRIEF-SR* Behavior Rating Inventory of Executive Function Self-Report, *Brixton* Brixton Spatial Anticipation Test, *CANTAB ID/ED* Intra/Extradimensional Set Shift subtest of the Cambridge Neuropsychological Automated Battery, *CCI* Central Coherence Index, *CIA* Clinical Impairment Assessment, *D-KEFS* Delis-Kaplan Executive Function System, *DFlex* Detail and Flexibility questionnaire, *EDQoL* Eating Disorder Quality of Life scale, *FPT* Fragmented Pictures Task, *GEFT* Group Embedded Figures Test, *IIP-32* Inventory of Interpersonal Problems, *KWCST* Wisconsin Card Sort Test Keio version, *KWCST DMS* Wisconsin Card Sort Test Keio version Difficulty Maintaining Set, *MFFT* Matching Familiar Figures Test, *ROCFT* Rey-Osterreith Complex Figure Task, *ROCFT OCI* Rey-Osterreith Complex Figure Task Order of Construction Index, *SF-36* Short Form Health Survey, SF-36 PCS Short Form Health Survey Physical Component Summary, *SF-36 MCS* Short Form Health Survey Mental Component Summary, *TMT* Trail Making Test, *ToM* Theory of Mind, *WCST* Wisconsin Card Sort Task, *WCST CC* Wisconsin Card Sort Task Categories Completed, *WCST PE* Wisconsin Card Sort Task Perseverative Errors, *WCST %PE* Wisconsin Card Sort Task percentage Perseverative Errors. See Tables 2 and 3 for details of included measures

**Table 2:** Amended Down’s and Black Quality Index scores for included studies

	***Reporting***					***Power***		
	**Aim described?**	**Main outcomes described?**	**Participant characteristics described?**	**Main findings described?**	**Estimates of random variability?**	**Probability values reported?**	**Power calculation?**	
Oldershaw et al. [60]	✓	✓	✓	✓	✓	x	x	
Bentz et al. [9]	✓	✓	✓	✓	✓	✓	x	
Hamatani et al. [36]	✓	✓	✓	✓	✓	x	x	
Westwood et al. [94]	✓	✓	✓	✓	✓	✓	x	
Renwick et al. [67]	✓	✓	✓	✓	✓	✓	x	
Talbot et al. [79]	✓	✓	✓	✓	✓	✓	x	
Calderoni et al. [14]	✓	✓	✓	✓	✓	✓	x	
Harrison et al. [38]	✓	✓	✓	✓	✓	✓	x	
Spitoni et al. [75]	✓	✓	✓	✓	✓	✓	✓	
Herbrich et al. [39]	✓	✓	✓	✓	✓	✓	✓	
Van Noort et al. [89]	✓	✓	✓	✓	✓	✓	x	
Stedal & Dahlgren [76]	✓	✓	✓	✓	✓	x	x	
McAnarney et al. [54]	✓	✓	✓	✓	✓	✓	x	
	***External validity***			***Internal validity***				
	**Invited participants representative of population?**	**Final participants representative of population?**	**Staff and facilities representative of population?**	**“Data dredging” made clear?**	**Statistical tests appropriate?**	**Measures valid and reliable?**	**Adjustment for confounds?**	**Total**
Oldershaw et al. [60]	✓	✓	✓	✓	✓	✓	✓	**12**
Bentz et al. [9]	✓	?	✓	✓	✓	✓	✓	**12**
Hamatani et al.	✓	✓	✓	✓	✓	✓	✓	**12**
Westwood et al.	✓	?	?	✓	✓	✓	✓	**11**
Renwick et al. [67]	✓	?	✓	✓	✓	✓	✓	**12**
Talbot et al. [79]	✓	✓	✓	✓	✓	✓	x	**12**
Calderoni et al.	✓	?	✓	✓	✓	✓	✓	**12**
Harrison et al. [38]	✓	?	✓	✓	✓	✓	✓	**12**
Spitoni et al. [75]	✓	✓	✓	✓	✓	✓	x	**13**
Herbrich et al. [39]	✓	?	?	✓	✓	✓	✓	**12**
Van Noort et al.	✓	✓	✓	✓	✓	✓	✓	**13**
Stedal & Dahlgren [76]	✓	?	✓	✓	✓	✓	x	**10**
McAnarney et al.	✓	?	?	✓	✓	✓	✓	**11**

### Quality assessment

Methodological quality of the included studies was assessed using a modified version of the Downs & Black Quality Index [23]. Items excluded from the original index were items relating to randomized controlled trials, as revised by Ferro and Speechley [28], and one item relating to description of response rate which were not useful assessment criteria for the current review. The amended 14-item index is presented in Table 2. Items were scored 1 for Yes (✓), and 0 for No (x) or Unable to determine (?). Maximum score was 14. The overall methodological quality of the studies included in the review was high. Two items were not well fulfilled; only two studies included a power calculation and less than half included information regarding the proportion of participants vs. those who were invited to participate.

### Sample characteristics and measures

Participants in the studies included in the review were demographically heterogeneous. Most studies included only female participants, one study included two males [79] and two did not specify [39, 60]. Five studies included only adult participants and six studies describe their cohorts as children, adolescents or young people. One study included participants 16–42 years [75], and one included participants 12–47 years [94]. Eight studies included a healthy control comparison group.

Participants were also clinically heterogeneous. Most studies included only participants with AN; one study also included participants with Bulimia Nervosa, however clear subgroup analysis was provided [38]. Eight studies identified their participants by diagnostic subtype or included only one subtype. Studies included participants across a spectrum of stage and severity of AN, and included inpatient and outpatients cohorts. Studies varied in their exclusion criteria, however all excluded participants with other serious conditions, e.g. neurological or developmental disorders. Most studies did not exclude participants using psychopharmacological medication.

Power is an issue for AN research. Five studies had a sample size of under 25 participants; four of these were studies which included only children and/or adolescents, one was an adult cohort [36]. Some studies where analysis was undertaken by subtype or stage of illness also had low numbers of participants per group.

Studies included a variety of measures of set-shifting and central coherence, summarized in Table 3. Neurocognitive predictor measures were all performance-based, except the Detail and Flexibility Questionnaire (DFlex). The Wisconsin Card Sort Task (WCST) was the most common set-shifting measure, used in nine studies (however, note [67] use as a measure of general executive function). The Rey Osterreith Complex Figure Test (ROCFT) Central Coherence Index (CCI) was the most common measure of central coherence, also used in nine studies.

**Table 3:** Cognitive measures in included studies

*Assessment*	*Type*	*Brief description*
**Set-shifting**		
Brixton Spatial Anticipation Test (Brixton; [13])	Performance	Participant must learn and apply rules to guess where a shape will appear next based on feedback from preceding trials.
Delis-Kaplan Executive Function System (DKEFS; [19]) Color-word interference test-4	Performance	Participants required to name the color ink in which a list of color names is printed (as Stroop task).
D-KEFS: TMT-4	Performance	As Trail Making Test below.
D-KEFS: Verbal fluency-3	Performance	Participant asked to say words from two designated semantic categories as quickly as possible while switching between categories.
Detail and Flexibility Questionnaire (DFlex; [68])	Self-report	24 items assessing cognitive rigidity and attention to detail in daily life.
Intra/Extradimensional Set Shift (IED) subtest of the Cambridge Neuropsychological Automated Battery (CANTAB) [71]	Performance	Participants must learn and apply rules guided by feedback. *Intra-dimensional shifts* - applying previous rule to a new stimulus set, are followed by *extra-dimensional shifts* - applying a new rule which was previously irrelevant. Rule changes after six consecutive correct responses.
NEPSY-II Attention and EF: Auditory response set, Inhibition shifting [44]	Performance	*Auditory response set*: Participants required to switch between matching (select blue circle when “blue” is said) and contrasting (select red when “yellow” said) response to verbal instruction. *Inhibition shifting*: Participants switch between responding to color or shape of bivalent stimuli.
Trail Making Test (TMT-A, TMT-B; [12])	Performance	TMT part B assesses shifting ability. Participant must draw lines to connect 25 circles containing numbers (1–13) and letters (A-L) in ascending order while alternating between numbers and letters (1-A-2-B) without lifting pencil. Timed.
Wisconsin Card Sort Test (WCST; [32])	Performance	Participants sort a target card to a stack of cards which match the color, shape or number of the target card guided only by feedback. Category (usually) changes every 10 trials.
**Central Coherence**		
Fragmented pictures task (FPT; [74])	Performance	Computerized task where a picture forms from fragments, frame by frame until participant correctly identifies the picture.
Group embedded figures test (GEFT; [98, 99])	Performance	Timed task where participants need to find simple shapes embedded in a more complex figure.
Matching Familiar Figures Test (MFFT; [41, 42])	Performance	Timed task where the participant must select the figure which matches a familiar object from an array of eight very similar figures.
Rey Osterreith Complex Figure Task (ROCFT; [61])	Performance	Participants copy a complex geometric figure. Central coherence index is calculated based on the order (global or local elements) and style of construction (continuous or fragmented).

Studies included a variety of functional outcome measures, summarized in Table 4. Six studies included measures of social function, three of which were social cognition measures. Six studies used measures designed to assess executive function in daily life. Two studies assessed ED-specific functional impairment and two studies measured quality of life. Functional outcome measures in five studies were self-report only. Two studies included a parent-reported measure and two included a clinician-reported measure. Four studies included a performance-based function measure; three of these were the tests of social cognition and one was a measure of executive function in daily life.

**Table 4:** Functional outcome measures in included studies

*Assessment*	*Type*	*Brief description*
Autism Diagnostic Observation Schedule (ADOS; [51])	Observer-rated	Semi-structured assessment where participant is observed in social interaction, communication, play and imaginative use of materials.
Behavioral Assessment of Dysexecutive syndrome (BADS; [97])	Performance	Six performance tests which assess everyday problem-solving: Rule Shift Cards test (shifting), Action Program test (problem-solving), Key Search (problem-solving), Temporal Judgement, Zoo Map (planning), Modified Six Elements (time-management).
Behavior Rating Inventory of Executive Function self-report (BRIEF-SR; [31])	Self-report	Assesses executive function impairment for participants aged 11–18 years. Two summary scores: *Behavior Regulation Index* (Inhibit, Shift and Emotional Control subscales) and *Metacognitive Index* (Initiate, Working Memory, Plan/Organize, Organization of Materials and Monitor subscales) and a *Global Executive Composite Score* (summary of all subscales).
Behavior Rating Inventory of Executive Function Parent Form (BRIEF-PF; [31])	Other-report	Assesses executive function impairment for participants aged 5–18 years.
Clinical Impairment Assessment (CIA; [10])	Self-report	16-items assessing psychosocial impairment secondary to ED symptoms. Global score and 3 subscale scores: personal, social and cognitive.
Eating Disorders Quality of Life (EDQoL; [25])	Self-report	25-item ED-specific health-related quality of life scale. Subscales: psychological, physical/cognitive, financial and work/school.
Inventory of Interpersonal problems (IIP-32; [6])	Self-report	32-items assessing interpersonal difficulties; things that are “too hard” to do and things done “too much” in relationships.
NEPSY-II [44] Social Perception: ToM verbal, ToM contextual and Affect recognition	Performance	*ToM verbal*: Participants answer questions requiring understanding of the point of view of a character in a verbal/pictorial vignette. *ToM contextual*: Participants match emotions to social situations. *Affect recognition*: Participants identify basic emotions in facial expressions.
Reading the Mind in the Eyes Test [7]	Performance	Participant views the eyes of facial photos only and selects a description of the emotion expressed from four options.
Short-Form Health Survey (SF-36; [91, 92])	Self-report	36-items. *Physical health summary*: physical functioning, limitations due to physical health, pain, general health. *Mental health summary*: vitality, social function, limitations due to emotional health, mental health.

### Are poor set-shifting and central coherence associated with everyday function in anorexia nervosa?

Overall there were more associations between set-shifting and functional outcome measures than between central coherence and functional outcome measures. Seven studies reported a direct or indirect association between a set-shifting and a function measure, and three studies reported an association between a central coherence measure and a function measure. Interpretations based on grouping these associations by neurocognitive measures are very limited due to the wide range of functional outcome measures used, therefore results are grouped according to functional outcome.

### Social function measures

Three studies used social cognition tasks to measure social functioning. In an adult sample with current AN, Harrison et al. [38] report higher WCST perseverative errors were associated with lower emotion recognition on the Reading the Mind in the Eyes test, however the association was not significant after Bonferroni correction.

Renwick et al. [67] tested adult participants with current AN on cognitive (WCST, Brixton Spatial Anticipation Test, ROCFT) and social cognitive (Reading the Mind in Films; RMIF) tests and used discriminant function analysis to identify three clusters representing a social-cognitive profile of high, mixed and poor performance. The cluster with poor overall performance is noted to have increased perseverative errors on the WCST, poor ROCFT copy trial scores and poor RMIF emotional theory of mind, which is interpreted as an indirect association between poor set-shifting, weak central coherence, and poor social-cognitive function.

Calderoni et al. [14] tested children/adolescents with AN-R using the full NEPSY-II battery which includes two shifting tests in the domain of attention and executive function, and three social perception tests. Direct associations between the set-shifting and social cognition tests were not included, however there were no differences between the AN-R patients and a healthy control group on the social perception tests (verbal theory of mind, contextual theory of mind and affect recognition). There were also no group differences on the overall domain of attention and executive function or the test of inhibition shifting, however performance on the response set-shifting test was marginally worse in the AN-R group.

Two studies used the Autism Diagnostic Observation Schedule (ADOS) as a measure of social functioning. Bentz et al. [9] tested an adolescent/young adult cohort comprised of individuals with first episode AN and those recovered from adolescent AN. A composite set-shifting score included the shift conditions from three Delis-Kaplan Executive Function System (D-KEFS) subtests: verbal fluency, design fluency and trail making. Composite set-shifting scores did not predict social function according to the ADOS total scores.

Westwood et al. [94] used ADOS Social Affect and Restricted and Repetitive Behavior scores to allocate a mixed age (12–47) cohort of participants with current AN into high vs. sub-clinical vs. no Autism Spectrum Disorder (ASD) symptoms groups. Participants were also tested on the WCST and the ROCFT. An increase in the percentage of perseverative errors on the WCST was associated with increased ASD symptoms, but the ROCFT was not associated with increased ASD symptoms.

One study used the Inventory of Interpersonal Problems (IIP-32). Talbot et al. [79] tested an adult cohort across stages of AN, including currently ill, weight-restored and fully recovered participants. A number of associations between WCST perseverative error (PE) scores and the IIP-32 are reported, however they are in the opposite direction to what may be expected. WCST PE scaled scores (where higher scores indicate better performance) were associated with higher scores on IIP-32 scores (which indicate greater interpersonal problems), in the subscales ‘Too dependent’, ‘Too aggressive’, ‘Hard to be involved’ and ‘Hard to be supportive’. Talbot et al. [79] also report two further associations in the opposite direction expected, between higher WCST Categories Completed (CC) and higher IIP-32 ‘Too dependent’ and ‘Too aggressive’. However, WCST CC is a measure of runs of 10 correct trials (when rule changes after 10 trials) and may be more indicative of general abstract reasoning, or the ability to learn how to ‘play the game’ [48]. Participants also completed the ROCFT, which demonstrated some relation to the IIP-32 in the predicted direction. Lower ROCFT Central Coherence Index and Order of Construction Index (OCI) were associated with greater endorsement of IIP-32 ‘Hard to be supportive’, and lower OCI was associated with higher IIP-32 ‘Hard to be involved’.

### Measures of executive function in daily life

Six studies used assessments of executive functioning in daily life. Four studies utilized the self–report version of the Behavior Rating Inventory of Executive Function (BRIEF-SR) in younger cohorts, and two of those also included the parent form (BRIEF-PR).

Herbrich et al. [39] found no group differences between children/ adolescents with AN and healthy controls for the WCST, ROCFT or the Group Embedded Figures Test (GEFT). Participants with AN-R were faster on the Trail Making Test (TMT) than controls. BRIEF-SR scores for both AN subtypes and controls were all within normal range, however scores on the “Shift” subscale were graded, with AN-R participants indicating more issues with shifting than the controls, and AN-BP participants indicating more problems than AN-R.

van Noort, Kraus, Pfeiffer, Lehmkuhl, and Kappel [89] tested adolescents with AN and healthy control participants on the TMT, ROCFT and BRIEF-SR before and after Cognitive Remediation Therapy. There were no group differences for TMT or ROCFT at baseline. Irrespective of time point, participants with AN reported less flexibility than controls with medium-high effect on the BRIEF-SR subscales “Cognitive shift” and “Behavioral shift”.

Stedal and Dahlgren [76] tested adolescents with AN with the ‘Ravello Profile’ battery which includes the Brixton, ROCFT, and the shift trials from the Delis-Kaplan Executive Functioning System (D-KEFS) TMT, colour-word interference and verbal fluency tests. Participants also completed the BRIEF-SR, and their parents completed BRIEF-PF. Scores for all performance-based test scores and the BRIEF-SR and BRIEF-PF were within normal range.

McAnarney et al. [54] tested adolescents and young adults with AN-R (14–20 years) on the WCST, CANTAB Intra-Extra Dimensional Set Shift Test (IED), BRIEF-SR and BRIEF-PF. Participants with AN-R scored more poorly than a control group on WCST total errors, but perseverative error scores were not significantly worse. There were no significant group differences on the IED. Self and parent-reported difficulties with shifting were greater for AN-R participants than controls. At the subscale level, differences between AN-R participants and controls were greater for the BRIEF-SR Behavioral Shift than BRIEF-SR Cognitive Shift.

One study tested a mixed age cohort of participants with AN using the BADS and the WCST, TMT and ROCFT. Spitoni, Aragonaa, Bevacqua, Cotugno, and Antonucci [75] found perseverative errors on the WCST were higher for participants with AN than a control group, though the effect size was small. Performance on the TMT-B was slower than controls with small to medium effect. Participants with AN had a lower ROCFT Central Coherence Index (CCI) than controls with small to medium effects at 30 s and 20 min recall, however, the direct copy trial is the preferred CCI measure [46], and there was no group difference on direct copy trial performance. On the BADS battery, participants with AN performed similarly to controls in accuracy, however AN participants were slower than controls across most tasks with medium to very large effect sizes.

One study used the Detail and Flexibility Questionnaire (DFlex), a self-report measure of cognitive rigidity and attention to detail in daily life. Westwood et al. [94] found no significant difference in DFlex scores across high vs. sub-clinical vs. no ASD symptom groups, however on the cognitive rigidity subscale scores were significantly higher (more rigidity) in the high than the no ASD symptoms group.

### Clinical impairment assessment

Two studies used the Clinical Impairment Assessment (CIA) to assess functional impairment secondary to ED symptoms. Renwick et al. [67] administered the CIA to an adult cohort with AN alongside the WCST, Brixton, and ROCFT (and social cognition measures, as described above), however a discriminant function analysis found no effect of cluster on CIA scores. Also in an adult cohort, Oldershaw et al. [60] found cognitive tests (WCST, Brixton, TMT, GEFT) did not predict CIA scores.

### Quality of life measures

Two studies used QoL measures. In a cohort of adults with AN, Hamatani et al. [36] found greater Difficulty Maintaining Set (DMS) on the WCST Keio version was associated with lower scores on SF-36 Physical Component Summary (PCS). However, PCS scores for AN participants were not significantly different from a healthy control group, and DMS is not a standard set-shifting measure. DMS is the number of times an incorrect response occurs after 2–5 consecutive correct responses and may be more indicative of distractibility, as failing to maintain set has been inversely associated with performance on a vigilance task [29]. In the same group of participants, ROCFT central coherence index 30-min delayed recall was positively associated with SF-36 Mental Component Summary, however, as mentioned previously, the direct copy trial is considered to be best measure [46].

Talbot et al. [79] report an association between higher WCST Categories Completed (CC) and better financial QoL. However, as mentioned above, WCST CC is not a standard set-shifting measure and may be more indicative of general abstract reasoning or the ability to learn the task-rule.

## Discussion

The current review synthesizes the existing evidence of associations between tests of set-shifting and central coherence and function measures in AN. Overall, the review highlights that this question has received little empirical focus, and of the studies which have assessed the question directly or indirectly, only a limited number of associations have been identified. Differences in the methodologies, measures and samples in the included studies complicate interpretation of the results, however it is possible to make a few general conclusions.

Firstly, the impact of set-shifting and central coherence deficits on functional outcomes in AN appears to be limited, and specific in nature. Levels of functional impairment have been compared to schizophrenia [4], however in schizophrenia neurocognitive deficits appear to exert a substantial general effect on overall functional outcome. The pattern of effect in AN represented by the current review is very different; associations between the cognitive tasks and functional outcomes were inconsistent, with many studies finding no association, and those noted were mostly at the subscale level, indicating a specific impact.

A second general conclusion is that the associations between set-shifting and central coherence deficits and functional outcome identified by the current review were in the adult samples, not the child/adolescent samples. There was generally little evidence of set-shifting and central coherence deficits on performance measures in the younger samples, which is largely consistent with meta-analytic results [40, 47]. However, significant differences between adolescents with AN and control participants were identified by the Behavior Rating Inventory of Executive Function (BRIEF).

Which is the third general conclusion; results of the current review suggest assessments of executive functioning in everyday life may be more sensitive to the cognitive-behavioral flexibility issues experienced by individuals with AN. This is particularly clear for the studies with child/adolescent participants where there were no group differences on the cognitive performance measures, but those that utilized the BRIEF identified that young people with AN report more difficulties with shifting in their daily lives than controls. These differences were noted using both the self-report and parent-report versions of the BRIEF. Results also suggest that behavioral shifting may be more problematic than cognitive shifting. This is consistent with neuroimaging research that suggests rigidity in AN may be secondary to impairments in behavioral response shifting rather than cognitive set-shifting [101].

Additionally, the one adult study which used a measure of executive functioning in everyday life, the Behavioral Assessment of Dysexecutive Syndrome (BADS), was the only study to find a general effect across measures. The BADS is comprised of tasks which are more complex and less structured than standard cognitive tests, involving not only the execution of a task, but planning, organizing, initiation and decision-making that is more similar to real-life task completion. Spitoni et al. [75] found adults with AN were just as accurate, but generally slower across most tasks, and concluded this may be due to perfectionism related to a detail-oriented processing style. The results are also consistent with a large synthesis of studies which questioned the nature of this general ‘slow-down’ effect seen in ED cohorts, which showed speed of information processing in simple reaction-time tasks was not impaired, but choice reaction time was slower [27].

Both the BRIEF and the BADS are test batteries designed to improve the ecological validity of executive function testing, an issue that has been extensively debated in the clinical and general population literature (see [16] for a review). Researchers in the eating disorder field have called for the use of more ecologically-valid measures to assess cognitive functioning in everyday tasks [15, 62, 76]. Objective performance tests with a specific focus on ecological validity have not been widely utilized in ED research, however measures of self-reported executive function are increasingly used, including ED-specific measures such as the Detail and Flexibility Questionnaire (D-flex; see Table 2), and a measure which encompasses both general and ED-specific flexibility, the Eating Disorder Flexibility Index (EDFLIX; [17]).

Although the current review suggests the nature of the impact of set-shifting and central coherence issues on functional outcome is specific, it is unable to identify which area of function is most affected. The review identified more associations with social function than other areas, however, it is not possible to conclude that social function is most impacted because many of the included studies were more focused on social difficulties than work or everyday activities. A focus on interpersonal problems is valid; patients with AN often experience reduced motivation for, and pleasure from, social interaction, and social-emotional avoidance is a key maintenance factor in the Cognitive-Interpersonal Maintenance Model for AN [72]. However, in terms of the question of possible associations with everyday function, investigations in other disorders suggest a focus on social impact may be an issue. Although results in schizophrenia suggest a general effect of neurocognitive performance on functional outcomes, there is a greater impact on occupational than social function [34]. And in obsessive-compulsive disorder, a more specific relationship has been identified; a retrospective in-patient study found poor set-shifting, measured by the Trail Making Test part B predicted poorer vocational outcome, but not social outcome or independent living status [64]. This raises the question: Could the use of outcome measures that are heavily weighted towards social items mean that the potential impact of cognitive difficulties is being missed by the outcome measure? Answering this question will require further systematic assessment of everyday function across social, workplace and everyday activities.

Although the quality of the studies included in the current review was generally high, the quality of the evidence regarding associations between the cognitive tests and functional outcome measures may be limited by several measurement and sample issues. For the majority of the included studies, associations with functional outcomes was not the primary outcome measure, therefore these issues are discussed not as criticisms of the studies, but rather to highlight methodological issues which may be addressed in future research.

### Measurement issues

Studies included in the review primarily used the Wisconsin Card Sort Task (WCST) as the set-shifting measure. Nine studies used the WCST, and in three cases [36, 79, 94] the WCST was the only set-shifting measure. Seven studies reported an association between a set-shifting and a function measure, and in five cases the measure was the WCST. This may be an issue, because the WCST has a limited ability to isolate set-shifting ability from other areas of executive function, known as the *task impurity problem*. The problem arises because performance on even a simple behavioral task will involve cognitive processes outside the variable of interest [56]. Executive function broadly encompasses three areas: *shifting* flexibly between mental sets, *updating* working memory, and *inhibition* of a prepotent response [57]. The WCST dependent variable for set-shifting ability is number of perseverative errors, or errors in sorting where enough information to derive the correct sorting rule has been given. However, perseverative errors could occur due to difficulties with monitoring or updating working memory or inhibiting a response. In particular, the ability to inhibit a previously rewarded response, or reversal learning ability, has been raised as a problematic confound for ED research which uses the WCST [96]. Overall, these issues mean the WCST is best taken as a general measure of executive function, as it was used in one included study [67]. Concurrent measurement of working memory and inhibition, or a composite set-shifting measure to reduce task-related variability may improve the measurement of shifting-specific variance. One study included in the review [9] used a composite measure comprised of the shift conditions of verbal fluency, design fluency and trail making tests from the D-KEFS.

Included studies primarily used the Rey Osterreith Complex Figure Test (ROCFT) as the central coherence measure. Nine of the twelve studies which assessed central coherence used the ROCFT, and in six studies the ROCFT was the only central coherence measure [36, 67, 75, 76, 89, 94]. To provide a more comprehensive assessment of central coherence, utilizing both a global processing measure and a detail-oriented processing measure to demonstrate a bias away from global and towards local processing is useful. Three studies reported an association between a central coherence measure and a function measure, in all cases the measure was the ROCFT, however only one of these studies calculated central coherence index using the direct copy trial, which is the preferred measure [46].

A wide variety of functional outcome measures were used in the included studies and differences in their sensitivity to detect the impact of cognitive inefficiencies may account for the inconsistent findings. A meta-analysis of the associations between cognitive tests and everyday function in Bipolar Disorder notes the strength of correlations varied more due to functional measurement than cognitive domain [20]. General measures may be designed for populations where deficits are of greater magnitude or are more global in nature, and may therefore lack sensitivity to the level or pattern of impairment experienced by individuals with AN. Disorder-specific function measures such as the CIA may be more sensitive to functional impairments than general measures, however significant associations were not found using the CIA in this review. Null results may be partly explained by the general finding that associations between performance-based and self-report measures are low [52, 76], and most included studies used performance measures of set-shifting and central coherence, and many used self-report measures of functional outcome.

Four studies included a performance-based function measure, however, three of these were tests of social cognition only. The Reading the Mind in the Eyes test is a common test of social cognition, however it may be more strongly related to vocabulary than to emotion recognition or theory of mind ability [59].

### Sample issues

Null effects may also be due to small sample sizes, or to the heterogeneity of the sample. Sample sizes in AN research are often low, and therefore it is usually only possible to avoid one of these issues. Significant differences in set-shifting and central coherence have been suggested between adults and children, by AN subtype, and between currently ill and recovered individuals, therefore investigating these groups separately is important. However, the power to detect effects was limited in the studies included in this review which sampled only children and/or adolescents, or split their analyses by diagnostic subtype. One study included a particularly wide age range of 12–47 years [94] which may be an issue because although maximum development in executive function occurs between 7 and 12 years old [65], executive function continues to develop until the early 20s, and age has been associated with performance on set-shifting and detail processing tasks [43]. However, age was included as a covariate in the analysis, and the WCST was the primary measure of set-shifting, on which performance is estimated to reach an adult level by around 12 years [65].

Finally, it is important to measure depression, anxiety and IQ (or an estimate of premorbid IQ) alongside cognitive measures. Evidence of the extent to which these factors affect performance on cognitive tasks is somewhat inconsistent in the general psychiatric literature, but results of a review in AN suggest set-shifting performance is negatively impacted by depression [2]. Most studies measured depression and anxiety, however three did not [75, 76, 79]. One study measured depression, but not anxiety [54]. An estimate of premorbid IQ was reported in all but one study [60].

There are also strengths and limitations to the current review. The database search included published conference abstracts and was supplemented by a dissertations and theses search. However, unpublished data and data not available in English was not included, and the review was not pre-registered. Although this may have biased the results, many of the studies included in the review report non-significant findings. To maximize the breadth of studies included and provide a rich analysis of the existing evidence in this area, the review included a broad range of functional outcome measures. However, differences in the methodologies employed in the included studies limit the conclusions which can be drawn from the review.

Extending beyond the scope of the current review, there may be issues related to the limited focus on set-shifting and central coherence deficits when investigating the possible impact of cognitive predictors on functional outcomes. A focus on these two deficits is not without reason - these are the areas where performance difficulties are consistently noted for participants with AN. However, the result is a very narrow measurement of potentially determining factors which does not consider the effects of areas of cognitive strength, or the possibility of compensatory strategy use. For example, individuals with AN show superior performance to control groups in verbal fluency and verbal category switching [77, 80], and verbal memory has been shown to be a strong predictor of everyday function in schizophrenia [34]. Data from one of the studies included in the current review showed performance on set-shifting and central coherence tasks did not predict ADOS social function – but higher verbal memory scores did [9]. Tests of specific cognitive processes may underestimate the ability to function in daily life if strong compensatory strategies like these are used [53]. Recent research found adolescent participants with weight-restored AN showed superior performance to a control group on a verbal and visual-motor set-shifting task, but when inhibition was controlled for in the analyses, their performance was worse, demonstrating strong inhibitory control was used as a compensatory mechanism [93]. Results such as this highlight that to understand the impact of neuropsychological tests on everyday life, a cognitive profile of not only deficits, but also the strengths which could compensate for them is needed. Research in this area could provide support for future cognitive strengths-based approaches to support functional recovery in AN, as has recently been suggested for first-episode psychosis [3].

## Conclusion

Despite increasing interest in the assessment of possible links between cognitive impairments and clinical symptoms in AN, very little empirical work has been undertaken to explore whether there is relationship with everyday function. The current review synthesized research which has noted direct or indirect associations between set-shifting and central coherence tests and functional outcome measures in AN across a broad range of samples, measures and methodologies. The results of the review suggest that poor set-shifting and central coherence as measured by performance on standard cognitive tests has only a limited and specific effect on functional outcome, but that measures of executive functioning in daily life may be more sensitive to functional difficulties experienced by individuals with AN. Although the quality of the included studies was high, functional outcome was often a secondary measure, and associations between cognitive performance and functional outcome have not been as systematically assessed as is evident in other psychiatric disorders. However, as mentioned by Green [33], although cognitive research ‘looks inwards’, the studies included in this review also attempt to ‘look outwards’ to understand how cognitive inefficiencies impact functional outcomes for individuals with AN. This is important, because functional improvements are milestones of recovery which may increase patient engagement with therapy in AN. Future research in this area could improve current cognition-focused therapies by identifying broader therapeutic targets, and functional milestones by which the therapies can be evaluated.

## Data Availability

Not applicable.

## Abbreviations

ADOS: Autism Diagnostic Schedule
AN: Anorexia Nervosa
AN-BP: Anorexia Nervosa Binge-Purge subtype
AN-R: Anorexia Nervosa Restricting subtype
ASD: Autism Spectrum Disorder
BADS: The Behavioral Assessment of Dysexecutive Syndrome
BMI: Body Mass Index
BRIEF: Behavior Rating Inventory of Executive Function
CC: Categories Completed
CCI: Central Coherence Index
CIA: Clinical Impairment Assessment
CRT: Cognitive Remediation Therapy
DKEFS: Delis-Kaplan Executive Function System
DMS: Difficulty Maintaining Set
DFlex: Detail and Flexibility Questionnaire
ED: Eating Disorder
GEFT: Group Embedded Figures Test
IED: Intra-Extra Dimensional set shift test
IIP: Inventory of Interpersonal Problems
IQ: Intelligence Quotient
OCI: Order of Construction Index
PCS: Physical Component Summary
PE: Perseverative Errors
PRISMA: Preferred Reporting Items for Systematic Reviews
QoL: Quality of Life
TMT: Trail Making Test
ROCFT: Rey Osterreith Complex Figure Test
WCST: Wisconsin Card Sort Task
